# Communication interventriculaire ischémique: à propos d'un cas observé dans le service de cardiologie du CHU-Yalgado Ouedraogo de Ouagadougou (Burkina Faso)

**DOI:** 10.11604/pamj.2014.19.342.5702

**Published:** 2014-12-02

**Authors:** Nobila Valentin Yaméogo, Maurice Ilboudo, Arthur Seghda, Jonas Kologo, Georges Millogo, Boubakar Jean Yves Toguyéni, André Samadoulougou, Patrice Zabsonré

**Affiliations:** 1 Service de Cardiologie, Centre Hospitalo Universitaire Yalgado Ouédraogo, Ouagadougou 03, Burkina Faso; 2 Université de Ouagadougou, UFR en Sciences de la Santé, Ouagadougou, Burkina Faso; 3 Centre Hospitalo Universitaire Yalgado Ouédraogo, Service de Chirurgie Générale

**Keywords:** Infarctus du myocarde, rupture myocardique, choc cardiogénique, Myocardial infarction, myocardial rupture, cardiogenic shock

## Abstract

La rupture myocardique est une complication rare mais souvent fatale de l'infarctus du myocarde aigu récent. Une patiente âgée de 72 ans, présentant une douleur thoracique typiquement angineuse évoluant depuis 34 jours, en insuffisance cardiaque globale était reçue pour une exploration cardio-vasculaire. L'examen physique retrouvait un souffle holosystolique endapexien d'intensité 3/6, irradiant en rayon de roue. La troponine T était élevée à quatre fois la normale et l'ECG objectivait une lésion sous épicardique en antéroseptoapical et une nécrose dans le même territoire. L’échodoppler cardiaque retrouvait un anévrisme septoapicolatéral avec une solution de continuité dans le segment apical du septum interventriculaire (CIV). Traitée par énoxaparine, antiagrégant plaquettaire, diurétique de l'anse, dérivés morphiniques et oxygène, la patiente présente au deuxième jour de son hospitalisation un collapsus cardio-vasculaire et décède dans un tableau de choc cardiogénique malgré l'administration des amines vasopressives à forte dose. La coronarographie n'a pu être réalisée. Ce cas illustre la gravité des complications mécaniques de l'infarctus du myocarde. L'absence de chirurgie cardiaque dans notre pays explique en grande partie l’évolution fatale de cette CIV ischémique.

## Introduction

La rupture myocardique après un infarctus du myocarde (IDM) aigu peut intéresser la paroi libre du ventricule gauche (ou droit), le septum interventriculaire et/ou les muscles papillaires. La survenue de chacun de ces événements est très rare et de pronostic très sombre [1]. Nous présentons l'observation d'une patiente de 72 ans, victime d'un infarctus en antérieur compliqué d'une rupture apicale du septum interventriculaire.

## Patient et observation

Madame Y F, âgée de 72 ans, ayant comme facteur de risque cardio-vasculaire l’âge, une hypertension artérielle non suivie et une obésité androïde, a été adressée pour la réalisation d'un ECG et d'un écho Doppler cardiaque afin de documenter une insuffisance cardiaque. L'anamnèse retrouvait une douleur thoracique typiquement angineuse, inaugurale, survenue depuis 34 jours et traitée dans les structures sanitaires périphériques par des pansements gastriques, des antalgiques et des anti-inflammatoires non stéroïdiens. L'examen physique retrouvait une tension artérielle à 100/70 mmHg, l'IMC à 30,85 kg/m2, le périmètre abdominal à 104 cm, un syndrome d'insuffisance cardiaque globale et un souffle holosystolique endapexien d'intensité 3/6, irradiant en rayon de roue. Les pouls périphériques étaient bien perçus.

**Figure 1:**
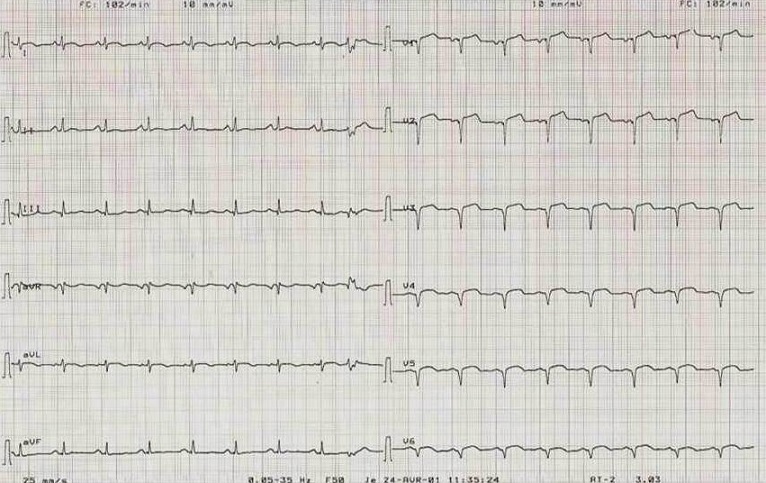
ECG à 12 dérivation montrant une lésion sous épicardique en antérieur étendu avec nécrose dans le même territoire

**Figure 2:**
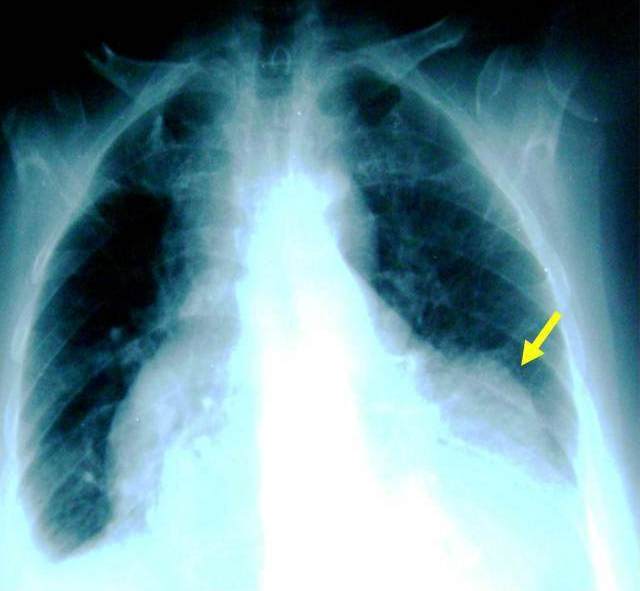
Télécoeur de face. Cardiomégalie (RCT = 0,75) avec excroissance de la portion inférieure de l'arc inférieur gauche du cœur (flèche)

**Figure 3:**
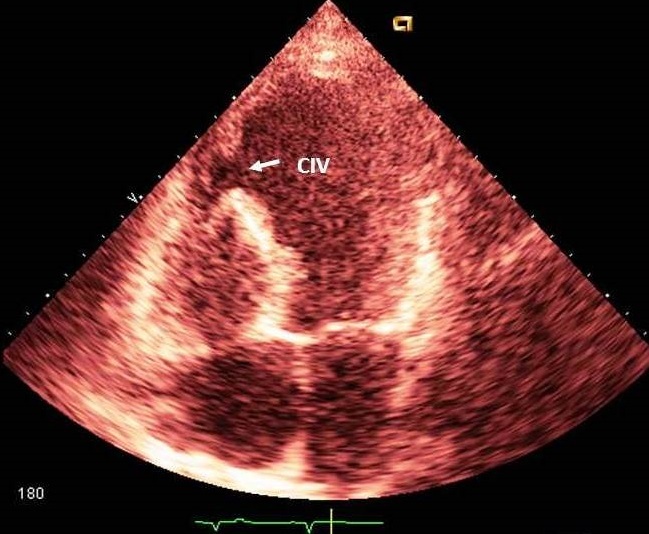
échocardiogramme transthoracique, coupe apicale incidence 4 cavités montrant la CIV (flèche)

**Figure 4:**
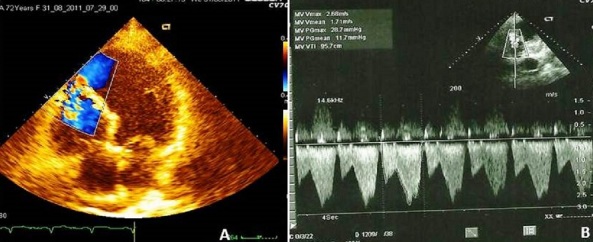
A) échocardiogramme transthoracique, coupe apicale incidence 4 cavités, doppler couleur montrant le flux de la CIV; B) échocardiogramme transthoracique, coupe apicale tronquée, doppler continu montrant un gradient interventriculaire moyen (à travers la CIV) de 11,7 mmHg et maximal de 28,7 mmHg

A la biologie, la troponine I était élevée à quatre fois la normale et les ASAT à 5 fois la normale. La glycémie, l'ionogramme sanguin, l'hémogramme et la créatininémie étaient normaux. L’électrocardiogramme de surface (Figure 1) s'inscrivait en rythme sinusal régulier avec une lésion sous épicardique en antéroseptoapical et une nécrose dans le même territoire. Il n'y avait pas d'extension au ventricule droit. Le téléthorax (Figure 2) objectivait une cardiomégalie (RCT = 0,75) avec une excroissance de la portion inférieure de l'arc inférieur gauche du cœur (flèche). L’écho Doppler cardiaque (Figure 3 et Figure 4) retrouvait un anévrisme septoapicolatéral avec une solution de continuité dans le segment apical du septum interventriculaire. Le traitement était constitué d’énoxaparine d'antiagrégant plaquettaire, de diurétique de l'anse, de dérivés morphiniques et d'oxygène. Au deuxième jour de son hospitalisation, la patiente présente un collapsus cardio-vasculaire qui a motivé l'introduction des amines. Elle décèdera malgré tout le lendemain dans un tableau de choc cardiogénique. La coronarographie n'a pas été réalisée.

## Discussion

La rupture myocardique est une complication redoutable de l'infarctus du myocarde. En effet, beaucoup de patients décèdent immédiatement à la phase préhospitalière sans aucune confirmation de la cause du décès de telle sorte que l'incidence globale est difficile à évaluer [2–4]. La rupture myocardique a été largement documentée avant l’ère de la revascularisation myocardique précoce par des études anatomiques et surtout échographiques [5], Farcot JC et coll. [6]. Mais il existe peu d’études depuis l'avènement de la thrombolyse préhospitalière et l'angioplastie primaire. Braunwald et coll. ont décrit 13 facteurs de risque de la rupture myocardique ischémique parmi lesquels l’âge avancé, le sexe féminin, le caractère transmural de l'infarctus et le territoire de l'interventriculaire antérieure [7]. Notre patiente présentait toutes ces caractéristiques. La rupture myocardique est à l'origine de 15% environ des décès à la phase aiguë d'un IDM récent [8]. Elle se manifeste en moyenne 3 à 10 jours après l'infarctus du myocarde, en général par un choc cardiogénique réfractaire conduisant au décès rapide avant même d'avoir pu bénéficier d'une réparation chirurgicale, dans la majorité des cas [9, 10].

A l’ère préthrombolytique l'incidence rapportée de la rupture septale était de 11% des séries anatomopathologiques et 2% des patients hospitalisés pour infarctus du myocarde; elle n'est plus que de 0,2% de nos jours [7]. La rupture septale apparaît plus volontiers dans les infarctus de localisation antérieure (66% des ruptures septales). C'est le cas de notre patiente. Elle intéresse en général la portion apicale des infarctus antéroseptaux avec nécrose transmurale. L’échocardiographie réalisée dès l'apparition du souffle permet de faire un diagnostic de certitude avec la mise en évidence du défect lui-même en mode bidimensionnel et du shunt en Doppler couleur [7]. L’échocardiographie par voie transthoracique permet de faire le diagnostic dans environ 80% des cas. Elle peut éventuellement être complétée par la voie transœsophagienne chez les patients peu échogènes. Les deux facteurs pronostiques principaux de la rupture septale sont l'importance de la dysfonction systolique globale secondaire à l'infarctus et l’étendue du défect septal, dont la manifestation clinique principale est l'instabilité hémodynamique. Le pronostic reste encore aujourd'hui très sombre avec une mortalité à 30 jours de l'infarctus à 74% [10]. Le traitement du défect septal est chirurgical et consiste en une fermeture par patch prothétique. Idéalement, le patient est proposé au chirurgien dans des conditions hémodynamiques optimisées par une prise en charge réanimatoire adaptée (ballon de contre-pulsion intra-aortique, agents inotropes positifs), dans les plus brefs délais [10]. Chez notre patiente, malgré l'usage des inotropes positifs, l’état hémodynamique est resté précaire jusqu'au décès.

## Conclusion

La rupture myocardique est une complication mécanique majeure de l'infarctus du myocarde. Elle engage le pronostic vital et nécessite des mesures de réanimation adaptées avant l'acte chirurgical. Le diagnostic est échographique. Le retard diagnostic est un facteur de mauvais pronostique. Les moyens de réanimation limités dans nos conditions d'exercice grève ce pronostic.
